# Psychometric properties of the Child Oral Impacts on Daily Performances (C-OIDP) index: a cross-sectional and an intervention study of adolescents in Bosnia and Herzegovina

**DOI:** 10.1186/s12903-023-03161-1

**Published:** 2023-06-28

**Authors:** Jelena Erić, Ljiljana Bjelović, Svjetlana Janković, Bojana Davidović, Djordje Bozović, Jelena Krunić

**Affiliations:** 1 Department of Prosthodontics, Faculty of Medicine, University of East Sarajevo, Studentska bb, 73300 Foca, Bosnia and Herzegovina; 2 Department of Dental Pathology, Faculty of Medicine, University of East Sarajevo, Foca, Bosnia and Herzegovina; 3 Department of Pediatric Dentistry, Faculty of Medicine, University of East Sarajevo, Foca, Bosnia and Herzegovina

**Keywords:** Oral health, Quality of life, Validity and reliability, Caries

## Abstract

**Background:**

The clinical measures are not sufficient to assess oral health because they don’t tell us anything about functional and psychosocial aspects of oral health and do not reflect person’s concerns and subjectively perceived symptoms. This study aimed to investigate the validity, reliability and responsiveness of the child Oral Impacts on Daily Performances (C-OIDP) index among Bosnian 12-14 years old schoolchildren.

**Methods:**

The study population comprised 203 primary schoolchildren aged 12-14 years attending three schools in the eastern part of Bosnia and Herzegovina. Data were collected through: a clinical oral examination, oral health questionnaire and C-OIDP questionnaire. The validity and reliability of the C-OIDP were tested on a sample of 203 school-going children while responsiveness of the C-OIDP was assessed on 42 randomly chosen participants requiring a dental treatment.

**Results:**

In terms of reliability, Cronbach’s alpha coefficient and the intraclass correlation coefficient were 0.86 and 0.85, respectively. Regarding the testing of construct validity, the C-OIDP score was increased as children’s self-reported oral health changed from excellent to very bad and from very satisfied to dissatisfied. There was a significant improvement in C-OIDP post-treatment score compared with C-OIDP pre-treatment score. Overall, 63.4% of participants reported at least one oral impact in the last 3 months. The most affected performances were “eating” (38.4%) and “speaking” (25.1%).

**Conclusion:**

The Bosnian version of the C-OIDP showed satisfactory validity, reliability and responsiveness and can be used as an appropriate OHRQoL measure for further epidemiological researches.

## Background

Oral health is an integral part of general health and enables persons to perform essential functions such as: breathing, eating and speaking, and encompasses psychological dimensions such as self-esteem and the ability to communicate with other people without any discomfort and embarrassment [1]. According to this concept, the measurement of oral health must include much more than clinical indicators [2]; the clinical measures are not sufficient to assess oral health because they don’t tell us anything about functional and psychosocial aspects of oral health and do not reflect the oral health status, functioning of a person as a whole, his/her concerns and subjectively perceived symptoms [3]. Therefore, great attention has been focused on oral health- related quality of life (OHRQoL) over the last decades and OHRQoL instruments have been using widely in conjunction with clinical indicators to assess the functional, social and psychological outcomes of oral disorders [4].

In literature, plenty of OHRQoL measures have been developed among adults and elderly population to assess the oral impacts on people’s day-to-day functioning. However, only a few OHRQoL indices are specifically designed to assess the OHRQoL in the child-adolescent population due to a different methodological and conceptual approach [5–7].

The Child Oral Impact on Daily Performance (C-OIDP) is one of the OHRQoL questionnaires that was widely used in different studies of children and adolescent populations [8–14] and found to be a valid and reliable measure to assess the impacts of oral condition on children’s everyday activities. Despite being used in many countries, the responsiveness of the C-OIDP in different countries have not been established. For this reason, it’s not clear if a questionnaire proved to be valid and reliable in cross-sectional studies will necessarily be appropriate for assessing the outcomes of clinical intervention. According to the literature [15, 16], longitudinal studies involving OHRQoL indices sought to measure changes in scores from baseline to post-treatment and determine the effect of treatment on OHRQoL. De Oliveira and Sheiham [16] found that Brazilian adolescents who had orthodontic treatment had better OHRQoL than their non-treatment counterparts. For this reason, along with other clinical assessments, it is very important to find out if a questionnaire can be used for assessing the efficacy of treatment protocols from patients’ perspectives.

In 2012, this scale was translated and cross-culturally adapted for Bosnian adult population [17] through close collaboration with the developers of the OIDP at University College London and used for assessing the relationships between OHRQoL and clinical measures of oral function in a sample of older adults in Bosnia as well [18]. On the other hand, the C-OIDP was translated and adapted into Serbian language (one of three official languages in Bosnia and Herzegovina) [19], but so far, no validation study of the translated Bosnian child version has been conducted in a clinical or population-based sample in Bosnian children and adolescents. Furthermore, Bosnian schoolchildren and adolescents have the high prevalence and severity of caries [20] compared to their countreparts from Western European countries. For this reason, information on the OHRQoL of schoolchildren are very important to add to knowledge on dental caries by examining the extent of impact of oral status on children’s daily activities.

Therefore, this study aimed to investigate the validity, reliability and responsiveness of the C-OIDP index among Bosnian 12-14 years old schoolchildren.

## Methods

This cross-sectional survey was conducted on a sample of schoolchildren aged 12-14 years living in three municipalities of the Eastern part of Bosnia and Herzegovina during the period May-July 2018. Ten primary schools are located in the Eastern part of Republica Srpska, Bosnia and Herzegovina, and three primary schools were randomly selected using the opaque envelopes [21]. The envelopes contained allocation to location of primary schools. The sample for the study was primarily selected in order to address the assessment of reliability and validity of the C-OIDP among schoolchildren with all permanent teeth in Bosnia and Herzegovina. Also, the C-OIDP was culturally adapted for use among 11–14 years old schoolchildren, and thus it was tested on the similar aged group. Regarding the minimum required sample size, we followed the rule of participants-to-item ratio for exploratory factor analysis (EFA) [12]. According to the EFA, the recommended participants-to-item ratio range from 5 to 20 participants per item [22, 23]. In this study, we used a 20:1 participant-to-item ratio to derive a minimum sample size of 160. Furthermore, in order to allow a 10% non-response rate, the minimum required sample size was 176 participants. A sample size of 100-200 participants was also recommended in the literature [24] for assessing validity and reliability of OHRQoL questionnaires.A total of 210 schoolchildren were invited to participate in the study but 7 of them didn’t give their consent or were absent. Therefore, a sample size of 203 was chosen for this study. This study was approved by the Institutional Ethics Committee of the University of East Sarajevo (No 01-8/71) and written informed consents were obtained from the parents of all the schoolchildren participated in the study.

### Pilot study

Before the main study, a pilot study was carried out on 42 children of the same age to assess the face and content validity of the child-OIDP questionnaire in Serbian speaking area. All necessary changes were made, including minor modifications of the wording to ensure clarity and understanding of the questionnaire [19]. For example, when the participants were asked the first question: “whether or not problems with mouth and teeth have caused any difficulty with …(performance)…?”, they couldn’t understand it well. For this reason, wording modifications were made to ensure clarity of the question. Furthermore, the order of questions was changed and children were first asked the fourth question of the original version of the C-OIDP index as it was recommended by author [8]. They were given a list of common oral problems occurring in this age group. So, the use of the questionnaire was much easier. This pilot study demonstrated that there were not content or conceptual differences between the original version and the backward translation of the questionnaire.

### Main study

Data were collected through: a brief clinical oral examination, oral health questionnaire and C-OIDP questionnaire.

The clinical oral examinations were carried out in dental school clinics or classrooms under florescent lighting by one trained and calibrated dentist according to the criteria described by the World Health Organization (WHO) [25]. The clinical oral examinations were used to determine the oral health status (caries, gingival status and type of occlusion). Caries was diagnosed based on visual-tactile criteria (using a mouth mirror and an explorer) determining the decayed, missing, filled teeth (DMFT) index. The DMFT was calculated as the sum of decayed, missed and filled teeth, and scores were categorized into those who caries-free (DMFT=0) and having caries (DMFT≥1). The Löe-Silness gingival index [26] was used to grade gingival status. The gingiva surrounding the six indexed teeth was evaluated at four sites: mesio-facial papilla, facial marginal gingiva, disto-facial papilla and lingual marginal gingiva. Gingival index (GI) of the individual was obtained by adding the values of each tooth and dividing by the number of selected teeth. The gingival tissues were assessed based on the following criteria: GI score of 0 indicated no signs of gingivitis, 0.1-1.0 mild gingivitis, 1.1-2.0 moderate gingivitis and 2.1-3.0 severe gingivitis. The scores were dichotomized into those having healthy gingiva (GI≤1) and those with gingivitis (GI>1) [13]. Malocclusion was assessed according to the Angle’s classification and children were divided into two groups: children with normal occlusion (Angle class I) and children with Angle’s Class II, and III malocclusions [27].

The self-administered questionnaire was included data on socio-demographic information, oral health habits, perceived oral health conditions and satisfaction with oral health. The following socio-demographic variables used in the study were: child’s gender (male vs female), child’s age (12-14), living area (urban vs rural), father’s educational level (low, medium, high), mother’s educational level (low, medium, high) and socio-economic status (low, middle). The measure of self-reported oral health habits included four questions: tooth brushing frequency (regular vs irregular), dental flossing (≥ twice a day or ≤ once a day), dental attendance (regular dental check-up vs symptom oriented) and sugar-sweetened snack intake (high consumer vs low consumer). The following three questions were included in the self-administered questionnaire as well: perceived oral health treatment need, self-rated oral health and satisfaction with oral health. The interview contained a single item question with two possible answers for “perceived oral health treatment need” (yes or no), five possible answers for “self-rated oral health” (very bad, bad, fairly good, good and excellent) and four possible answers for “satisfaction with oral health” (not satisfied, partly satisfied, satisfied, very satisfied).

The C-OIDP index was used to assess the OHRQoL of schoolchildren. It measures oral impacts that affect different daily life performances: eating, speaking, cleaning teeth, sleeping, smiling, emotional state, studying and social contacts. Participants were asked to assess the frequency and severity of oral impact on the daily life using three-point ordinal scales (0-3), with zero score if no impact was reported. If a child reported that he/she had impact on any daily activities for more than 3 months, 3 points were assigned, for more than 1 up to 3 months 2 points were assigned and for one, two days up to a month 1 point was assigned. Also, using a scale from 0 to 3, where 0 is no effect and 3 a very severe effect, they had to respond how much effect the difficulty has had on their everyday life [19]. The response scores were: 0 (no effect), 1 (a fairly minor effect), 2 (a moderate effect) and 3 (a fairly severe effect). Performance scores were calculated by multiplying the frequency and severity scores for each C-OIDP item, while the total score was obtained as the sum of eight performance scores divided by 72 (the maximum possible score) and then multiplied by 100. Higher C-OIDP scores represent poorer OHRQoL.

Eventually, responsiveness of the C-OIDP was tested on 42 randomly chosen participants requiring a dental treatment such as: filling, root canal treatment, extraction or cleaning. After an initial examination, all the eligible participants’ names and the file numbers (188 of them) were placed in a jar and 42 of them was picked using a lottery method. At least 2 months after the treatment was given, the same group of children completed the C-OIDP questionnaire again. It was supposed that the OHRQoL would improve considerably within a 2-month period after the treatment, as compared with the pre-treatment status. Change scores for the C-OIDP as children’s perceptions of change in their oral health following dental treatment at the clinic were evaluated by a single item with a five-point scale: (1) worsened a lot; (2) worsened a little; (3) stayed the same; (4) improved a little; and (5) improved a lot.

### Data analysis

The SPSS software package, version 20.0 (IBM Corp., Armonk, NY, USA) was used for data analysis. Descriptive analyses were used to calculate frequency distributions and percentages of demographic and clinical data as well as the C-OIDP. The prevalence of each C-OIDP item was calculated as the number of participants who had difficulty with eating (speaking, cleaning etc.) divided by the total number of participants in the sample and multiplied by 100. The total prevalence was calculated by dichotomizing the scores to yield the categories (0) “no daily performance affected” and (1) “at least one daily performance affected”. The percentage of those with at least one daily performance formed the total prevalence of C-OIDP.The internal consistency of the C-OIDP was assessed using standardized Cronbach’s alpha, alpha if item deleted, inter-item and item-total correlation coefficients. Test-retest reliability was tested by the intra-class correlation coefficient (ICC) and weighted kappa, using data from 20 schoolchildren who were re-interviewed 2 weeks after the first visit. Construct validity was measured with non-parametric tests (Mann–Whitney or Kruskal–Wallis tests) using data from 203 schoolchildren to determine relationship between the C-OIDP score and subjective health status measures (perceived dental treatment needs, global self-reported oral health status and satisfaction with oral health). For discriminant validity, the C-OIDP score was compared with clinically assessed dental status, gingival status and malocclusion using data from 203 schoolchildren. At the review appointment (after at least 2 months), the participants were also asked to rate the global change in their condition on a global transition judgement scale. Change scores for the C-OIDP were assessed by a single item with a five-point scale ranging from “worsened a lot” to “improved a lot”. The answers were recorded into three categories: worsened (including the original categories “worsened a lot” and “worsened a little), remained the same (including the original category “stayed the same”) and improved (including the original categories “improved a little” and “improved a lot”). Firstly, the mean pre-treatment and post-treatment C-OIDP scores were calculated for those who reported “worsened”, “stayed the same” and “improved” and then the change score was calculated by subtracting post-treatment from pre-treatment scores for the total score and individual domains. Paired t-test was used to test the significance of the difference in the C-OIDP summary score between the baseline and the follow-up scores. Also, the effect size (ES) was estimated by dividing the mean of change score by the standard deviation of the pre-treatment score [28]^.^

## Results

A total of 210 schoolchildren were invited to participate in the study and 7 of them did not give their consent (response rate: 96.67%). There were 56.2% boys and 43.8% girls. Most participants 85.7% lived in the urban area and most of their parents had middle education level.

Clinically, the mean DMFT scores was 5.00 (SD=3.38). 47.3% of children had gingivitis of varying severity, 12.3% had malocclusion, 47.8% visited dentists regularly for check-ups, 88.7% brushed their teeth regularly, 43.8% used dental floss more than twice a day, and 59.6% were low sugar consumers (Table 1).

**Table 1:** Socio-demographic, clinical and behavioral variables of the participants (*n=*203)

**Characteristics**		***N* (%)**
Gender	Male	114 (56.2)
	Female	89 (43.8)
Area	Urban	174 (85.7)
	Rural	29 (14.3)
Father’s education	Low	26 (12.8)
	Medium	104 (51.2)
	High	73 (36.0)
Mother’s education	Low	20 (9.9)
	Medium	125 (61.6)
	High	58 (28.6)
Socio-economic status variable	Low	51 (25.1)
	Middle	152 (74.9)
Tooth brushing	Regular	180 (88.7)
	Irregular	23 (11.3)
Dental flossing	≥ twice a day	89 (43.8)
	≤ once a day	114 (56.2)
Dental attendance	Regular check up	97 (47.8)
	Symptom oriented	106 (52.2)
Sugar-sweetened snack intake	High consumer	82 (40.4)
	Low consumer	121 (59.6)
Caries	DMFT≥1	188 (92.6)
	DMFT=0	15 (7.4)
Gingival index	GI≤1	107 (52.7)
	GI>1	96 (47.3)
Malocclusion	Present	25 (12.3)
	Not present	178 (87.7)

The prevalence of oral impacts was high; 63.4% of Bosnian participants reported at least one oral impact on their daily life in the last three months. The most common OIDP impact was difficulty with eating, reported by 38.4% of Bosnian schoolchildren. The next most prevalent impacts on Bosnian schoolchildren were related to speaking (25.1%) and smiling (18.7%), while the prevalence with the lowest impact was “studying” (Table 2). The mean C-OIDP score was 13.79 (SD=16.13).

**Table 2:** Prevalence of oral impacts on daily performance in the study population (*n=*203)

**Performances**	***N* (%)**
Eating	78 (38.4)
Speaking	51 (25.1)
Cleaning teeth	33 (16.3)
Sleeping/Relaxing	23 (11.3)
Emotional status	25 (12.3)
Smiling	38 (18.7)
Studying	16 (7.9)
Social contacts	32 (15.8)
At least one of the above	128 (63.4)

In terms of internal reliability, the inter-item correlation coefficients among 8 items scores of the C-OIDP index for Bosnian schoolchildren ranged from 0.21 to 0.78. The majority of these correlations were statistically significant (Table 3).

**Table 3:** Reliability analysis: Inter-item correlation for the C-OIDP in the study population (*n=*203)

Performance	Eating	Speaking	Cleaning teeth	Sleeping	Emotional status	Smiling	Studying	Social contacts
Eating	1.00							
Speaking	0.50	1.00						
Cleaning teeth	0.50	0.58	1.00					
Sleeping/Relaxing	0.36	0.37	0.60	1.00				
Emotional status	0.26	0.58	0.40	0.21	1.00			
Smiling	0.35	0.71	0.41	0.31	0.78	1.00		
Studying	0.37	0.34	0.57	0.30	0.39	0.33	1.00	
Social contacts	0.33	0.56	0.54	0.40	0.62	0.73	0.58	1.00

All inter-item correlations were positive, indicating homogeneity among the items. The corrected item-total correlation coefficients ranged from 0.48 to 0.73 (Table 4). The standardized Cronbach's alpha coefficients was 0.86. These alpha values did not increase if any of the items were removed. In terms of test-retest reliability, the intraclass correlation coefficient (ICC) and weighted kappa on repeated applications of the measure were 0.85 and 0.87 respectively.

**Table 4:** Reliability analysis: Corrected item-total correlation, Cronbach's alpha and alpha if item delated

Performances	Corrected item-total correlation	Alpha if item deleted
Eating	0.51	0.87
Speaking	0.73	0.84
Cleaning teeth	0.70	0.84
Sleeping/Relaxing	0.48	0.87
Emotional status	0.63	0.85
Smiling	0.72	0.84
Studying	0.55	0.86
Social contacts	0.73	0.84
Cronbach's alpha coefficient		0.86

Regarding the testing of construct validity, the relationship between the C-OIDP index and both subjective measures (global self-rated oral health and oral satisfaction) were strongly significant (*p<*0.001) (Table 5). Similarly, children with perceived needs for oral treatment had much higher C-OIDP scores than those who did not think they needed treatment (*p<*0.001).

**Table 5:** Findings for construct and discriminant validity for C-OIDP in the study population (*n=*203)

**Variables**	**Categories**	**N (%)**	**C-OIDP score** Mean (SD)	***P***
Perceived oral treatment needs	Yes	96 (47.3)	4.01 (4.98)	*p<*0.0001^*^
	No need	107 (52.7)	1.08 (2.85)	
Global self-rated oral health	Very bad	31 (15.3)	10.00 (3.97)	*p<*0.0001^†^
	Bad	7 (3.4)	8.57 (6.03)	
	Fairly good	39 (19.2)	1.30 (2.18)	
	Good	87 (42.9)	0.71 (1.66)	
	Excellent	39 (19.2)	0.45 (1.01)	
Satisfaction with oral health	Not satisfied	13 (6.4)	7.62 (5.79)	*p<*0.0001^†^
	Partly satisfied	63 (31.0)	4.78 (5.24)	
	Satisfied	74 (36.5)	0.86 (2.08)	
	Very satisfied	53 (26.1)	0.71 (1.90)	
Dental status	DMFT=0	15 (7.4)	0.03 (0.13)	*p<*0.01^*^
	DMFT≥1	188 (92.6)	2.66 (4.36)	
Gingival status	GI≤1	107 (52.7)	0.44 (2.36)	*p<*0.001^*^
	GI>1	96 (47.3)	4.72 (4.73)	
Malocclusion	Present	25 (12.3)	5.02 (5.87)	*p<*0.01^*^
	Not present	178 (87.7)	2.11 (3.86)	

^*^Mann–Whitney test for associations of C-OIDP with: ‘perceived oral treatment needs’

^†^Kruskal–Wallis test for associations of C-OIDP with: ‘global self-rated oral health’ and ‘satisfaction with oral health’

In relation to the discriminant validity, children with caries (DMFT≥1) (*p<*0.01), gingivitis (GI>1) (*p<*0.001) and malocclusion (Angle class II and III) (*p<*0.01) reported a higher C-OIDP scores when compared their counterparts (Table 5).

Overall, there was a significant improvement in C-OIDP post-treatment score compared with C-OIDP pre-treatment score (mean C-OIDP scores before and after treatment: 13.79 and 4.17; *p<*0.001). 59.5% of participants reported improved oral health following treatment at the clinic, while 23.8% and 16.7% reported no change and worsened oral health, respectively. As shown in Table 6, the mean change score was predominately negative among those who had worsened oral health (*p<*0.05) and positive in participants reporting no change (*p<*0.01) and improved oral health (*p<*0.001). The ES for the scale estimated for all participants was 0.59.

**Table 6:** Distribution of change scores in C-OIDP by global self-rated perceptions of change

Participant’s perception	Number of subjects N (%)	Change score Mean (SD)	p^*^
worsened	7(16.7)	-0.59 (7.69)^a^	*p*>0.05
stayed the same	10 (23.8)	8.61 (5.84)^b^	*p<*0.01
improved	25 (59.5)	12.89 (13.37)^b^	*p<*0.001
all subjects	42 (100.0)	9.62 (10.21)^b^	*p<*0.001

^*^Statistical evaluation by paired t-test

^a^Negative change score for the children who reported worsened oral health

^b^Positive change score for the children who reported stayed the same and improved oral health

## Discussion

To our knowledge, this is the first study to assess the psychometric properties of the C-OIDP in Bosnian schoolchildren population. Cultural adaptation of the C-OIDP questionnaire was previously done during the pilot study and the comparison between the original version and the backward translation of the questionnaire did not show any content or conceptual differences. Also, the face and content validity of the questionnaire were confirmed in the pilot study before using the questionnaire in the main study [19].

The Bosnian C-OIDP index showed that it is a reliable and valid measure of OHRQoL. The internal consistency of the index in terms of reliability, inter-item correlation, corrected item- total correlation and Cronbach’s alpha was excellent. All inter-item correlations were positive and all item- total correlations were above 0.20 [29]. The Cronbach’s alpha was higher than the values obtained in other cultural settings [8, 9, 12–14], indicating excellent psychometric properties compared with the standard recommended level of 0.7 [30]. The Cronbach’s alpha values were described in the literature as excellent (0.9), good (0.8), acceptable (0.7), moderate (0.6), not satisfactory (0.4) and low (0.1) [31]. All the alpha values above 0.7 indicate an acceptable internal consistency [30]. Furthermore, the external reliability of index revealed that the value of ICC and weighted kappa were 0.85 and 0.87 respectively, indicated a good agreement. This is consistent with previous studies conducted in United Kingdom and Pakistan [8, 14].

The Bosnian version of C-OIDP showed good construct validity in this study. Investigating the relationship between C-OIDP score and subjective oral health measures, we found that the perceptions of oral health, satisfaction and treatment need were significantly associated with the C-OIDP score. Consistent with previous studies, if the perception of children about their oral health was better, the C-OIDP score was lower. Also, as in some other studies conducted in Italy [9] and Turkey [13], we used clinical measures in the validity testing. Bivariate analysis in this study revealed that the presence of dental caries, gingivitis and malocclusion was significantly associated with higher C-OIDP score, showing a clear trend in the expected direction. These findings are in line with those obtained in other validation studies [9, 13, 32].

The prevalence of oral impact on daily performance was high, as 63.4% of Bosnian participants reported at least one daily activity being affected in the last three months. This is similar to studies in Indonesia (64.9%) [11] and Italy (66.8%) [9], lower than in the studies in Turkey [13] and Pakistan [14], but higher than in some previous studies [8, 10, 12]. These differences may occur due to socio-cultural differences or different disease levels between the population evaluated. Also, it has been documented that cultural beliefs, values and practices may have an impact on the perception of oral health and influence the condition of the teeth and mouth, through diet, care-seeking behaviors, or use of home remedies [33]. Butani et al. [33] in their systematic review reported that Mexican Americans residing in various parts of the United States of America had no adequate knowledge either about the connection between oral health and consumption of sugary food and frequent snacking or the role of fluoride in caries prevention. Furthermore, the Chinese used traditional medicine such as drinking a cooling tea or taking herbal medicine for minor oral health problems.

The most impacted daily performances were “eating” and “speaking”, reported by 38.4% and 25.1% of the participants respectively. These results are in line with other studies conducted in Turkey [13], Nepal [10] and Pakistan [14], where “eating” was reported as the most prevalent difficulty. On the other hand, Arumrahayu et al. [11] found that “cleaning teeth” was the most impacted daily performance. Furthermore, consistent with previous study [13], we found that the least frequently reported performances were “studying” and “relaxing”. These differences in self-assessed oral impacts may occur as a result of different children’s perception of health and illness which are affected by age, the stage of development and cultural settings [13, 33].

Important step in the process of psychometric validation of the C-OIDP index is to test its responsiveness in order to detect change in oral health occurring naturally or as a consequence of intervention. In this study, we analyzed change in OIDP scores in a subsample that required and received dental treatment. The study showed that those with more extreme scores at baseline had less scores at the follow-up, except those who reported deteriorating. The mean change scores significantly declined after the treatment, for the total score and for individual domains, except those who reported deteriorating. Children who reported worsened oral health had negative change score, indicating deterioration in OHRQoL, while subjects who reported improved oral health had highest positive change score, showing the highest improvement in OHRQoL. These results can be explained by high C-OIDP pre-treatment score and high levels of dental caries (DMFT=5.0) among Bosnian schoolchildren. Also, this is similar to other studies [34, 35] demonstrating good responsiveness to clinical changes of the C-OIDP. On the other hand, some OHQoL indices such as Early Childhood Oral Health Impact Scale (ECOHIS) [36] showed limited responsiveness when applied to a population with low caries treatment needs. This finding was explained by low levels of dental problems detected in their sample at the baseline. In addition, the C-OIDP showed good longitudinal construct validity. It is expressed by the mean C-OIDP change scores showing a clear gradient in the expected direction across the global ratings of perceptions of change in oral health status. In comparison with other studies, the effect size was larger than the ES obtained with C-OIDP among Norwich [34], Swedish [34] and Tanzanian children [35].

This study has some limitations and strengths that have to be considered when interpreting the results. The study used a sample of 12-14 years old schoolchildren from three primary schools in three Serbian-speaking municipalities. For this reason, our findings are not representative for the entire children population in Bosnia and Herzegovina. Despite this limitation, this is the first time that the validity and reliability of the C-OIDP index was assessed among Bosnian children. Furthermore, this study investigated the responsiveness of the Bosnian C-OIDP to dental treatment in order to prove its appropriateness for assessing outcomes of treatment procedures and oral health educational programmes.

## Conclusion

This study showed that the Bosnian version of C-OIDP is a valid, reliable and responsive measure of OHRQoL.This measure can be used in further epidemiological studies in the country either for identifying children with poor OHRQoL or assessing the effectiveness of therapeutic procedures.

## Data Availability

The datasets used and analyzed during the current study are available from the corresponding author on reasonable request.

## Abbreviations

C-OIDP: Child Oral Impacts on Daily Performances
OHRQoL: Oral health-related quality of life
DMFT: Decayed, missing, filled teeth
WHO: World Health Organization
GI: Gingival index
ICC: Intraclass correlation coefficient
ES: Effect size
ECOHIS: Early Childhood Oral Health Impact Scale
